# The Marvelous in Medicine

**Published:** 1890-04

**Authors:** E. Van Goidtsnoven

**Affiliations:** Atlanta, Ga.


### THE MARVELOUS IN MEDICINE.

BY E. VAN GOIDTSNOVEN, A. M., M. D., ATLANTA, GA.

"They the truth
With superstitions and traditions taint,"
—[*Milton.*

Faith-cure, Christian science, Spiritualism, are but modern forms of witch-craft. The belief therein, the belief in the existence of an order of beings and of things different from those that fall under our senses, the belief in the possibility to enter into communication with them, to obtain from them knowledge which can only be acquired by study and observation, and to perform through their assistance or agency deeds which exceed the limits of our natural faculties—this belief, I say, has been that of every people, and has been adopted and nursed by every religion.

Mystical ideas and superstitious practices vary *ad infinitum.* All rest upon the same foundation. During the dark days of ignorance these ideas and these practices mingled with medicine, jurisprudence and the governments of States. They brought forth incalculable evils; and after the New Platonists introduced them into their philosophy, they for ten centuries opposed a barrier to the progress of science and knowledge. This, therefore, constitutes one of the principal phases in the history of the human mind, and an additional reason to seek for its origin, and to examine the order and series of proofs upon which it was thought and believed to rest.

When a mystic opinion is brought forth from the brain of one man, and upheld by a few enthusiastic followers, it is hardly worth discussing.

Vagaries and credulity are of constant occurrence. But when this opinion is found engraved in the history of every age, held by men of superior intelligence, when this opinion is established upon innumerable testimonies and upon faces related by people of known veracity, it deserves the attention of scrutiny; and although it may appear absurd, it should be weighed as though it were doubtful.

Far be it from me to regard the unanimous accord of people as convincing and irrefutable.

When Cicero used it as an argument, he undoubtedly applied it only to sentiments inspired by nature and to moral principles born of conscience. But this accord is an argument which must be refuted by solid reasonings, and not by contempt. Opinions contrary to physical laws are simply due to ignorance. The discovery of a principle or of a fact suffices to render their further maintenance untenable.

But whilst I regard certain things that are of the domain of the supernatural, as the offspring of a diseased imagination, and of an aberrated mind, yet I must admit it is not likely that future generations will be better guarded against this evil than those that have preceded us.

Ridicule does not eradicate errors. It but increases their secret propagation. Persecution gives them strength. Sorcery never had more adepts and devotees than when it was hunted down by law.

The triumph of truth is secured only by wise and sincere discussion, thoroughly backed by knowledge. In the investigation of the marvelous only those facts that are well authenticated should receive attention. Their natural causes are made possibly plain. They can be dissociated from their consequences, and from the agency which they have been attributed to.

This investigation is certainly worthy of the attention of the philosopher, the scientist, the physician.

